# Resistant Gram-Negative Bacteria and Diagnostic Point-of-Care Options for the Field Setting during Military Operations

**DOI:** 10.1155/2018/9395420

**Published:** 2018-06-12

**Authors:** Hagen Frickmann, Andreas Podbielski, Bernd Kreikemeyer

**Affiliations:** ^1^Department of Microbiology and Hospital Hygiene, Bundeswehr Hospital Hamburg, Bernhard Nocht Str. 74, 20359 Hamburg, Germany; ^2^Department of Medical Microbiology, Virology and Hygiene, University Medicine Rostock, Schillingallee 70, 18057 Rostock, Germany

## Abstract

The spread of multidrug-resistant bacteria in resource-poor settings affects the military medical service in case of deployments of soldiers to war and crisis zones. Patients with war injuries are prone to colonization or infection with multidrug-resistant bacteria. Resistant Gram-negative bacteria play a dominant role in military wound infections. Problematic hygiene conditions on deployment facilitate exposition of soldiers with subsequent colonization. Although colonizing strains are frequently cleared from their hosts after returning from deployment, transmission to close contacts of the soldiers in the home country cannot be excluded and therapeutic options are reduced if colonization progresses to invasive infection. Since sophisticated culture-based diagnostic approaches are typically not available in the field setting on deployment, molecular rapid diagnostic test systems are an option for transmission control if the locally prevalent molecular resistance mechanisms are known. Efforts for global resistance surveillance can contribute to better understanding of resistance distribution and spread at deployment sites. This review summarizes experience of the military medical services with multidrug resistance on deployment and with the influx of resistant strains to the home country and discusses potential use of available molecular rapid test systems as an option for the field setting.

## 1. Introduction

Traveling to resource-limited areas is associated with reversible but substantial changes of the human gut microbiome [1]. If this phenomenon is potentiated by the influences of traveler's diarrhea and consumption of antibiotic drugs, the enteric selection risk for multidrug-resistant bacteria increases tremendously [2]. Accordingly, multidrug resistant pathogens and especially Gram-negative bacteria are frequent colonizers of the gut of travelers returning from the tropics [2–5]. Treatment in sub-Saharan African healthcare facilities was shown to increase the colonization risk to more than 90% [6]. However, specific exposure risks beyond the healthcare setting are widely unknown so far [7].

Concomitant to the reversion of the gut microbiome to the pretravel status [1], enteric colonization with multidrug-resistant bacteria is usually reduced in the course of several months, with asymptotic dynamics of pathogen clearance [8]. However, 11.3% returned travelers remained colonized even 12 months after their return in a recent assessment [9] and the probability of ESBL transmission to household members was 12.0%.

**Figure 1:**
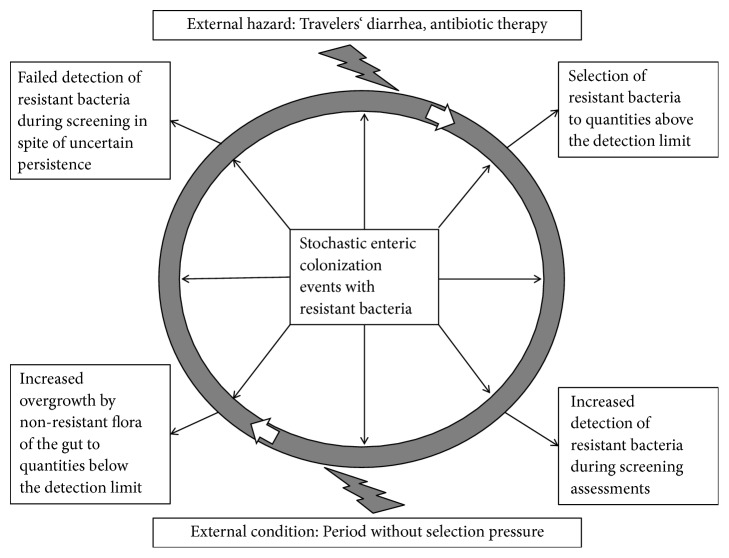
Examples of the influence of external conditions on the detection of enteric colonization with resistant bacteria by culture-based screening approaches [2, 8].

As screening for multidrug-resistant colonizers is still poorly standardized [10], it remains questionable what a negative screening result after previous colonization with resistant bacteria really means: either definite vanishing of the resistant pathogen or just a shift to a concentration below the detection limit. As preanalytic conditions like swabbing techniques [11] and the use of enrichment broths [12] were shown to relevantly affect the reliability of enteric screening approaches, it is highly likely that a proportion of individuals with apparently cleared colonization remains colonized with multidrug-resistant bacteria on a level below the diagnostic threshold. For these persons, there is a risk of selective enrichment of the multidrug-resistant bacteria under antibiotic pressure (Figure 1).

Similar to civilian travelers, soldiers deployed to resource-limited settings are exposed to a relevant acquisition risk of colonization or infections with multidrug-resistant bacteria [13]. In spite of a high likelihood of exposure, scientific literature on multidrug-resistant bacteria in deployed or returned soldiers is only scarcely available compared with our knowledge from civilian settings. Most of the assessments are published by US American or British authors. Recognizing the urgency of the issue, the US military early established a multidrug-resistance surveillance network [14]. The “Antimicrobial Resistance Monitoring and Research Program” was designed to allow for large-scale antimicrobial resistance surveillance [15].

But even prior to systematic surveillance approaches, military medicine had to deal with resistance problems from the beginning of modern antimicrobial therapy as occasionally documented in scientific literature. While early experience of the medical armed forces with antibiotic resistance in the last century was primarily focused on* Mycobacterium tuberculosis*,* Neisseria gonorrhoeae*, and Gram-positive methicillin-resistant* Staphylococcus aureus* (MRSA) [16–21], multidrug resistance in Gram-negative* Enterobacteriaceae* and Gram-negative rod-shaped bacteria is presently realized as an increasing menace.

In this narrative review, published experience of military medical services with Gram-negative multidrug-resistant bacteria is summarized and mobile, field compatible diagnostic systems are introduced.

## 2. Mode of Literature Review

Literature search was performed using the data bases NCBI PubMed https://www.ncbi.nlm.nih.gov/pubmed/, last accessed 4^th^ May 2018) and Google Scholar (https://scholar.google.de, last accessed 4^th^ May 2018) using the key words “military medicine”, “multidrug-resistance”, “molecular rapid testing”, “Gram-negative”, “rapid diagnostic test”, “ESBL”, “carbapenemase”, “Xpert”, “BioFire”, and “Amplex” in various combinations. Assessment of suitability for this narrative review was based upon the subjective impression of the authors.

## 3. Experience from Military Medical Facilities in Theater

Military conflicts are associated with an increased risk of distribution and spreading of multidrug-resistant bacteria. Influx of newly detectable strains of multidrug-resistant bacteria into crisis and war zones has been described. In the course of the Euromaidan riots in the Ukraine, a bla_NDM-1_-producing* Klebsiella pneumoniae* strain of the clonal complex ST11 was isolated for the very first time from a wound of an injured individual in this country [22].

However, traditional culture-based microbiology is difficult to maintain in deployment settings and thus rarely available in theater in crisis and war zones, especially in resource-limited settings. Therefore, data on microbial resistance of isolates from the deployment site are particularly scarce. Again, the resource-rich US American armed forces are an exemption. In order to identify extended spectrum beta-lactamase (ESBL) producing* Enterobacteriaceae* and other resistant bacteria, identification systems are provided in deployed laboratories by US forces [23].

The associated effort has led to providing a considerable set of data by the US military. In a study period between 2005 and 2007, 2,242 US casualties from Operation Iraqi Freedom and Operation Enduring Freedom were screened for multidrug-resistant bacteria. The three most frequently isolated pathogens comprised Gram-positive methicillin-resistant* Staphylococcus aureus* (MRSA) but also Gram-negative* Klebsiella pneumoniae* and* Acinetobacter* spp., each leading to nosocomial infection rates between 2% and 4% [24]. Interestingly, the overall detection rates for Gram-negative pathogens were much higher in locals in a role 3 medical facility (field hospital) in Iraq than in US forces irrespective of the sample material, while for Gram-positive bacteria, a prevalence inverse to the previously described was recorded [25].

High rates of multidrug resistance, in particular among Gram-negative organisms, were reported from war injuries during the recent conflicts in Iraq and Afghanistan [26]. In a recent point prevalence assessment from the European Union Training Mission in Mali (EUTM MLI), an enteric colonization rate of 27.1% (13 / 48) ESBL-positive* Enterobacteriaceae* could be demonstrated for European soldiers with traveler's diarrhea [27, 28].

*Acinetobacter* spp. are feared due to their complex resistance patterns, resulting in complex therapeutic regimens in the case of systemic infections, e.g., in primarily sterile compartments [29]. As early as during the 2003-2005 military operations of the US military in Iraq, predominantly osteomyelitis but also burn and deep wound infections with* Acinetobacter* spp. required complex antibiotic treatments for 6 weeks [30]. Carbapenem-resistance, which was frequently caused by* bla*_*Oxa-23*_ expression in* Acinetobacter* spp., was shown to be associated with prolonged stays in hospital and on intensive care units (ICU) of military treatment facilities [31]. In resource-limited deployment settings with restricted numbers of ICU beds, this can be problematic and even more so in the case of outbreak situations due to nosocomial transmission.

Factors affecting the risk of postsurgical wound infections of soldiers on deployment including those due to multidrug-resistant pathogens comprise a variety of elements including the presence of devitalized tissue, foreign bodies, blood clots, seroma, and contamination of wounds with bacteria from the casualties' skin, the environment, and the hospital itself [32]. Of note, the very early wound stages directly after infection are predominantly associated with susceptible strains as shown in a study with casualties in Iraq with only two out of 49 cases with MRSA detection and no proof of resistant Gram-negative flora [33]. This suggests transmission of multidrug-resistant strains in later stages of wound management in the military field medical care setting.

Due to the resistance-associated difficulties in antibiotic treatment, wound infections with carbapenem-resistant bacteria are particularly feared. To quantify the dimension of this problem, the US military medical service conducted an assessment of carbapenem-resistant* Enterobacteriaceae* prevalence in wounds of military personnel within a 6-years-period from 2009 to 2015. Fortunately, as few as 0.4% (16 out of 4090 strains) collected strains were carbapenem-resistant. The isolates most frequently comprised* Enterobacter aerogenes* (44%),* Klebsiella pneumoniae* (37%), and* Escherichia coli* (19%). In five strains from two patients, the responsible carbapenemase genes (4x* bla*_*KPC-3*_, 1x* bla*_*NDM-1*_) were successfully identified [34].

Caring for patients with multidrug-resistant pathogens is a risk of getting colonized and of further spreading these pathogens. An intensive patient contact of less than 30 minutes including endotracheal suctioning from a wounded US soldier without use of a surgical mask was shown to be sufficient to allow for transmission of multidrug-resistant* A. baumannii* to a healthcare worker as confirmed by molecular typing [35]. Such examples are suitable to underline the necessity for patient care in protective equipment if multidrug-resistant bacteria have to be expected, making the management of patients more complex and expensive.

## 4. Experience from Military Medical Healthcare Facilities in the Home Country

Acquisition of colonization with multidrug-resistant bacteria by soldiers on deployment consequently leads to an influx of resistant strains into military hospitals in the home countries where soldiers are treated in case of repatriation due to severe diseases or injuries. Next to this, healthy returnees from deployments are at risk of spreading colonizing multidrug-resistant bacteria among their families as previously shown for civilian travelers [9]. Fortunately, the earlier described phenomenon of spontaneous loss of ESBL-positive* Enterobacteriaceae* from the gut of civilian travelers after returning from the tropics [8] could be confirmed for soldiers. In a recent assessment of 828 German soldiers returning from deployments between 2007 and 2015, the average colonization rate with* Enterobacteriaceae* with resistance against third-generation cephalosporins was only 4.7% (39 / 828) 3 months after returning [36] while during tropical deployment colonization rates of up to 27% were observed in European soldiers with diarrhea [27, 28]. All isolates were* Escherichia coli* and ESBL was the most frequently detected resistance mechanism (37 ESBL, 1 ESBL + ampC, and 1 uncertain mechanism) [36]. In comparison, prevalence of* E. coli* with resistance against third-generation cephalosporins in the German population ranged between 5% to <10% in 2007 and 10% to <25% in 2015 in the assessment period as suggested by the European Centre for Disease Prevention and Control (ECDC, https://ecdc.europa.eu/en/antimicrobial-resistance/surveillance-and-disease-data/data-ecdc, last accessed 4^th^ May 2018). The distribution of ESBL-positive colonizing bacteria in the returned German soldiers differed by deployment site. In returnees from an UN-observer mission, where soldiers purchased their food on local markets and were exposed to the local hygiene conditions in this way, colonization rates up to 20% were observed [36]. In contrast, no* Enterobacteriaceae* with resistance against third-generation cephalosporins were isolated from samples from soldiers returning from nontropical deployment sites like Kosovo in the same assessment [36]. On average, however, colonization rates were similarly low and on the level of the home country 3 months after returning.

The asymptotic decolonization curve as suggested by Ruppé et al. [8], however, does not exclude transmission risks in the immediate term after returning from abroad. This topic was addressed by several studies which focused on prevalence in local military medical facilities in both the home countries or partner countries and abroad.

From a US military hospital in the home country, a longitudinal observation on the development of the spread of ESBL-positive* E. coli* and* K. pneumoniae* over a 7-years-period from 2003 to 2011 was described. From 2005 to 2010, ESBL incidence was moderately increased from low baseline levels for* E. coli* from 0.13% to 1.0% and for* K. pneumoniae* from 1.0% to 2.55% with predominance in females with urinary tract infections. Nearly half of the infections with ESBL-positive strains were not associated with comorbidities [37].

Between 2007 and 2011, the distribution of clonal lineages of ESBL-producing* E. coli* in US military service members in the home country was shown to resemble the distribution in other North American populations with dominance of ST10 (24%), ST131 (16%), and ST648 (8%). Clonal identity was also shown to be suitable to predict the most likely resistance pattern [38].

As shown in a report from 2015, the incidence of the particularly problematic* Enterobacteriaceae* with carbapenem-resistance in US military medical facilities was fortunately still as low as 1 per 100,000 patient years, although proportions differed among years, geographical regions, and bacterial species. Consumption of fluoroquinolones was shown to trigger the detection of carbapenem-resistant* E. coli* while no other significant associations could be demonstrated [39].

During a three-years period from 2009 to 2012, active screening-based surveillance for colonization with multidrug-resistant bacteria was conducted by US military at Landstuhl Regional Medical Center (Landstuhl RMC), Germany, and at three other regional treatment facilities. Colonization rate in Landstuhl was 6.6% and thus comparable with the local population in Germany. In comparison, it was nearly double as high (12.4%) at the three other facilities. Multidrug-resistant* E. coli* was most frequently identified, followed by* A. calcoaceticus*-*baumannii* complex and* K. pneumoniae*, without relevant quantitative changes over the assessment period [40].

Presently, the US screening efforts for multidrug-resistant bacteria were intensified and amended by whole genome next-generation sequencing. With multiple global sampling sites, the resulting strain collection comprises several 10,000 isolates [41].

The Trauma Infectious Disease Outcome Study [42] on deployment-related trauma in the period from 2009 to 2014 classified Gram-negative rod-shaped bacteria to be multidrug-resistant if resistance to ≥3 antibiotic classes or, alternatively, expression of extended spectrum *β*-lactamases (ESBL) or carbapenemases were observed. Based on this definition, a total of 26% (n= 245) military trauma patients with infections were affected by multidrug-resistant bacteria. The most commonly isolated species comprised* E. coli* (48.3%, n=262),* Acinetobacter* spp. (38.6%, n=210), and* K. pneumoniae *(8.4%, n=46). Risk factors for colonization with multidrug-resistant Gram-negative bacteria were severe trauma, comprising blast injuries and traumatic amputations. These data confirm that the association of war-related trauma and colonization or infection by multidrug-resistant bacteria is considerable.

The influx of multidrug-resistant bacteria with wounded soldiers also leads to secondary phenomena. As the risk of resistance has to be considered in case of calculated antibiotic therapy of severely ill patients, broad-spectrum antibiotic drugs have to be used [43] with the risk of additional selection of resistant pathogens.

Military conflicts also lead to an influx of multidrug-resistant bacteria to civilian hospitals of countries where care for transferred war-injured patients or refugees from crisis zones is provided. E.g., the prevalence of multidrug-resistant bacteria in war casualties from Libya transferred to a civilian tertiary hospital in Germany was assessed. In total, multidrug-resistant pathogens were detected in 60% of the patients. Carbapenem-resistant Gram-negative bacteria predominated (37%), but also Gram-positive MRSA (16%) was observed. Carbapenem-resistance was detected in* K. pneumoniae*,* A. baumannii*,* E. coli*,* Enterobacter cloacae*, and* Serratia marcescens* with* bla*_*NDM*_ (n = 17),* bla*_*OXA-48*_ (n = 15), and* bla*_*OXA-23*_ (n = 9) being the most frequently detected carbapenemase genes [44]. Multiple other studies with war-injured patients from the recent conflicts in Libya and Syria [45–53] showed comparable results.

In German military hospitals, intense colonization of patients with war injuries from crisis and war zones in Libya, Syria, and the Ukraine has been observed [54, 55]. Molecular analyses by rep-PCR and NGS suggested that nosocomial transmission within the military hospitals could be reduced to very low rates by the enforcement of strict hygiene precautions. Clonal identity of nonnosocomial strains, however, suggested transmission events either in medical facilities in the countries of origin or during evacuation flights under narrow spatial conditions. In spite of considerable efforts to achieve local decolonization by disinfectant washing, the results were only moderately better than the spontaneous decolonization rates. In addition, the effects depended on the compliance of the patients [56].

## 5. Diagnostic Point-of-Care Solutions for Potential Use on Deployment

Diagnosis of bacterial resistance in military deployment settings is challenging. Biochemical approaches like Microscan (Siemens AG, Munich, Germany) panels have been used to identify ESBL-positive* Enterobacteriaceae* in deployed laboratories of the US military [23]. However, culture-based resistance testing is laborious and difficult to provide on small missions in resource-limited settings.

In recent years, various molecular rapid diagnostic test (RDT) systems have been introduced for the identification of a number of quantitatively important resistant genes. The most frequently described molecular RDT systems for such purposes comprise PCR-based tools like the Xpert system (Cepheid, Sunnyvale, CA, USA) and the FilmArray system (BioFire Diagnostics, Inc., Salt Lake City, UT, USA), as well as loop-mediated-amplification-(LAMP)-based tools like the eazyplex system (AmplexDiagnostics GmbH, Gars Bahnhof, Germany) which will be described in more detail below.

While such molecular RDT tools are usually rapid and easy-to-apply, so the demand of skilled and highly qualified laboratory personnel can be reduced, they still require electrical power, maintenance, and appropriate transport logistics in the field. Another disadvantage is the fact that only the targeted resistance genes are detected. Therefore, the interpretability of their results largely depends on precise knowledge of the local resistance patterns and the underlying molecular mechanisms. In the case of multidrug-resistant Gram-negative pathogens, numerous resistance mechanisms may play a role while molecular RDT systems only detect the more frequent resistance genes. Accordingly, they are suitable for tracking a defined outbreak strain with a targeted resistance gene. However, a reliable exclusion of phenotypic resistance is not feasible in this way.

In detail, Public Health England recently compared three molecular systems for the detection of carbapenemases, i.e., the Check-Direct CPE kit (Check-Points BV, Wageningen, The Netherlands), the molecular RDT systems eazyplex SuperBug complete A kit (AmplexDiagnostics GmbH), and the Xpert Carba-R kit (Cepheid). All assays including the two RDT correctly identified all assessed strains with *bla*_KPC_, *bla*_VIM_, *bla*_NDM_, and classic *bla*_OXA-48_ carbapenemase genes while the coverage of other carbapenemase genes varied. The authors concluded that, among other factors, the preferred choice of gene coverage will be relevant for purchase decisions [57]. Several Xpert (Cepheid) systems were evaluated in various studies. While the Xpert MDRO (Cepheid) assay targets the carbapenemase genes* bla*_*KPC*_,* bla*_*NDM*_, and* bla*_*VIM*_ [58], the Xpert Carba-R assay (Cepheid) detects the carbapenemase genes* bla*_*IMP-1*_,* bla*_*KPC*_,* bla*_*NDM*_,* bla*_*OXA-48*_, and* bla*_*VIM*_ [59]. In 2015, however, French investigators had shown weakness of the Xpert Carba-R approach regarding the identification of* bla*_*OXA-48*_-like carbapenemase genes [60]. Consequently, the Xpert Carba-R v2 (Cepheid) was designed to allow the additional detection of* bla*_*OXA-181*_ and* bla*_*OXA-232*_ in addition to the spectrum of the Xpert Carba-R system [61].

However, a point of concern is the fact that the most systems are evaluated either with colonies which require prior culture-based growth or with mere screening materials like swabs from hygiene screenings. Accordingly, they are of uncertain reliability if a diagnosis directly from clinical sample material is desired and prior culture-based growth shall be avoided.

### 5.1. Evaluation of Molecular RDT Systems with Agar Cultures and Hygiene Swabs

While hygiene swabs can be used for surveillance purposes, the results of testing of such swabs showing mere colonization are not useful for the management of an individual patient. Nevertheless, such studies provide a first overview on performance characteristics of molecular RDT systems and are thus summarized in the following.

As an example of such evaluations from agar cultures, the eazyplex system (AmplexDiagnostics GmbH) correctly identified *bla*_OXA_ and *bla*_MBL_ carbapenemase genes in 82 nonrelated* Acinetobacter* spp. within less than 30 minutes per reaction [62]. Again, the importance of precise knowledge on prevalent local resistance mechanisms by active surveillance in the area of deployment has to be stressed for the interpretation of respective results.

Other studies were focused on hygiene swabs or stool samples, which provide epidemiological surveillance information but are not of use for clinical diagnosis. In 2013, an evaluation of the Xpert MDRO (Cepheid) assay targeting the carbapenemase genes* bla*_*KPC*_,* bla*_*NDM*_, and* bla*_*VIM*_ was published in comparison with culture with and without broth enrichment for rectal, perirectal, and stool samples. Sensitivity, specificity, and positive and negative predictive value were 100%, 99.0%, 93.0%, and 100% for* bla*_*KPC*_, respectively, and 100%, 99.4%, 81.8%, and 100% for* bla*_*VIM*_, respectively. No such statement could be made for* bla*_*NDM*_ due to lacking samples. In a serial dilution of stool samples spiked with a* bla*_*NDM*_-positive* K. pneumoniae* strain, 100% positivity at dilutions from 300 to 1,800 colony forming units (cfu) / ml and 93.3% at 150 cfu / ml were observed [58].

The Xpert Carba-R assay (Cepheid) targeting carbapenemase genes* bla*_*IMP-1*_,* bla*_*KPC*_,* bla*_*NDM*_,* bla*_*OXA-48*_, and* bla*_*VIM*_ showed a positive and negative agreement with culture and DNA sequencing as well as a positive and negative predictive value of 60%-100%, 98.9%-99.9%, 95%-100%, and 100%, respectively, when directly applied on rectal swabs [59]. Other authors suggested good performance of the system as well [63]. In a small Korean assessment, the Xpert Carba-R assay was more sensitive for the detection of carbapenemase-positive enteric colonization than culture [64].

In a small study with screening swabs from assumed high risk patients for carbapenemase-positive bacteria, 100% sensitivity, 99.13% specificity, 85.71% positive predictive value, and 100% negative predictive value were suggested for the Xpert Carba-R v2 system in comparison with selective culture [65].

### 5.2. Evaluation of Molecular RDT Systems with Clinical Sample Materials

Little data is available for the application of the Xpert systems with clinical sample materials. A recent Italian study stressed the importance of local epidemiology for the reliability of the Xpert Carba-R assay in a study with rectal/stoma swabs but also with swabs with abdominal drainage fluid from patients with abdominal sepsis, a material which is of potential interest for the management of severly ill patients. If only carbapenem-resistant bacteria carrying the targeted resistance genes were considered, sensitivity, specificity, and positive and negative predictive value of the Xpert Carba-R system were 100% (95% CI 69.1-100), 94.2% (95% CI 80.8-99.3), 83.3% (95% CI 59.6-97.9), and 100% (95% CI 89.4-100), respectively. If all carbapenem-resistant bacteria were considered, however, these values dropped to 50% (95% CI 24.6-75.3), 93.1% (95% CI 77.2-99.1), 80% (95% CI 44.4-97.5), and 77.1% (95% CI 56.9-89.6), respectively [66]. In another study using the Xpert Carba-R assay at least with spiked bronchial fluids, LOD was calculated to be < 10^4^ cfu/ml [67], providing some hints on analytical sensitivity with this important kind of clinical sample material.

Another device for potential use as an RDT in the field is the FilmArray system (BioFire Diagnostics, Inc., Salt Lake City, UT, USA). The FilmArray blood culture identification panel which was designed for rapid identification from positive blood culture materials also comprises three resistance genes (*mecA*,* vanA/B*, and* bla*_*KPC*_), including one (*bla*_*KPC*_) which occurs in Gram-negative pathogens [68–71]. In an eight-center trial with 2,207 positive aerobic blood culture samples in the USA, sensitivity and specificity were 100% for* vanA/B* and* bla*_*KPC*_ gene detection each and 98.4% and 98.3% for* mecA* gene detection, respectively [71]. In a South African study, consistency with the reference methods was even 100% for all tested resistance genes [70]. Although blood culture bottles are closed systems which are easy to handle, the assessment with the FilmArray blood culture identification panel nevertheless requires a cultural incubation step and is thus poorly suited as a real point-of-care approach.

Again, it has to be stressed that such molecular RDT approaches can detect the targeted resistance genes only, so results have to be interpreted with care regarding phenotypic resistance since other mechanisms cannot be excluded. Further, it is a major limitation that evaluation data of the introduced systems with clinically important primary sample materials are widely lacking, not allowing definite conclusions on the use of such systems as molecular RDT systems directly from clinical sample materials. So it remains widely unclear whether these assays show reliable results also directly from more complex sample matrices like blood, urine, or sputum or whether they will still require an initial culture-step before testing. If an initial culture-step is required, then the RDT platforms become less useful in the field environment. Suitable studies should be conducted either with spiked samples or with real clinical materials apart from just hygiene swabs with such systems to decide on their suitability for potential future use as stand-alone point-of-care solutions without the necessity of prior steps of culture-based growth.

## 6. Conclusions

Colonization and infection risks with multidrug-resistant bacteria are relevant issues for soldiers on deployment in high prevalence settings. This is particularly true for war injuries as shown for soldiers [42] and war-injured patients from different countries [54, 55].

Considering the fact that colonization frequently precedes infection, the observation by Yun et al. [25] that local forces in Iraq showed high colonization rates of skin and mucous membranes with Gram-negative bacteria is of particular importance. Similar data on Gram-negative colonization of skin and mucous membranes with Gram-negative* Enterobacteriaceae* were recently described for patients, students, and healthcare workers in the highlands of Madagascar [72]. The reasons are unclear, although high temperatures and humidity which are frequent in subtropical and tropical settings were described to facilitate Gram-negative bacterial growth on human skin [73].

Other sources of exposition include fecal contamination of food. As recently shown for the hotel canteen of the headquarters of the EUTM MLI mission in Western Africa, food contamination with ESBL-positive* Enterobacteriaceae* on deployment can occur when local hygiene conditions are poor [74].

If colonization with multidrug-resistant bacteria leads to infections on deployment, antimicrobial therapeutic options are scarce and prolonged cycles of combined antibiotic therapy become necessary [30]. Being aware of the fact that sophisticated resistance diagnostics are hardly achievable in remote conflict settings, nonspecific therapeutic approaches like silver-nylon dressings which are not prone to clinically relevant resistance selection are discussed [75]. Another option repeatedly discussed in the military medical service [76] is phage therapy as a potential alternative or at least an add-on to traditional antibiotic treatment.

The hygiene management of injured patients is also complicated by colonization or infection with multidrug-resistant bacteria, although nosocomial transmission in military medical facilities can be widely prevented if strict hygiene precautions are enforced [54, 55]. The effects of local skin or mucous membrane disinfection on decolonization of such sites are moderate in comparison to spontaneous decolonization rates and depend on the compliance of the patients [56].

If underlying resistance mechanisms are known, e.g., in the case of a local outbreak, molecular RDT systems might support the outbreak management in the field. However, such RDT systems target a restricted spectrum of resistance genes. Accordingly, they show poor sensitivity in case of nonspecific screening for phenotypic resistance as recently demonstrated [66].

If sophisticated diagnostic approaches in the field are not available in case of small deployments, knowledge of local prevalence and distribution of bacterial resistance is helpful for calculated antibiotic therapy in case of severe infections. To facilitate the efforts towards a global surveillance of multidrug-resistant bacteria, the US military service provides next-generation sequencing (NGS) capacities to provide a public database of collected strains from all over the world [41, 77]. As previously shown [78], this technology is suitable not only to show nosocomial transmission of strains but also on-site transmission of resistance genes between different bacterial species in wounds. Also, novel resistance-associated plasmids from remote war zones can be characterized [79]. In a similar way, the whole genome assessment also characterizes the distribution and spread of resistance genes and, thus, allows for association studies.

Multinational cooperation in the field of global resistance surveillance seems desirable to provide information on prevalence and spread of antimicrobial resistance worldwide, both for civilian and for military medical purposes.
